# Legume Intake Is Associated with Potential Savings in Coronary Heart Disease-Related Health Care Costs in Australia

**DOI:** 10.3390/nu14142912

**Published:** 2022-07-15

**Authors:** Mohammad M. H. Abdullah, Jaimee Hughes, Sara Grafenauer

**Affiliations:** 1 Department of Food Science and Nutrition, Kuwait University, Kuwait 10002, Kuwait; 2 Grains & Legumes Nutrition Council, 1 Rivett Rd, North Ryde, Sydney 2113, Australia; j.hughes@glnc.org.au; 3 Faculty of Medicine and Health, School of Health Sciences, University of New South Wales, Randwick 1466, Australia; s.grafenauer@unsw.edu.au

**Keywords:** legumes, coronary heart disease, health care cost, cost-of-illness analysis, nutrition economics

## Abstract

Legume intake has been associated with lower risk for a number of chronic disorders of high financial burden, and is advocated by dietary guidelines as an important part of healthy dietary patterns. Still, the intake of legumes generally falls short of the recommended levels in most countries around the world despite their role as an alternative protein source. The aim of this study was to assess the potential savings in costs of health care services that would follow the reduction in incidences of coronary heart disease (CHD) when adult consumers achieve a targeted level of 50 g/day of legumes intake in Australia. A cost-of-illness analysis was developed using estimates of current and targeted legumes intake in adults (age 25+ y), the estimated percent reduction in relative risk (95% CI) of CHD following legumes intake, and recent data on health care costs related to CHD in Australia. A sensitivity analysis of ‘very pessimistic’ through to ‘universal’ scenarios suggested savings in CHD-related health care costs equal to AUD 4.3 (95% CI 1.2–7.4) to AUD 85.5 (95% CI 23.3–147.7) million annually. Findings of the study suggest an economic value of incorporating attainable levels of legumes within the dietary behaviors of Australians. Greater prominence of legumes in dietary guidelines could assist with achieving broader sustainability measures in relation to diet, helping to bring together the environment and health as an important pillar in relation to sustainability.

## 1. Introduction

Legumes are increasingly the focus of discussions related to the future food supply due to their sustainability credentials and unique nutritional profile. Research suggests that although there is widespread promotion through food-based dietary guidelines, global intake patterns vary greatly. Whereas the term ‘legumes’ is the most inclusive word for the group of foods from the Fabacae (or Leguminosae) botanical family and is mentioned in 94 guidelines, fewer (n = 87) choose to depict legumes, beans, peas, or pulses only in the accompanying visual guide (unpublished data). Dietary guidelines act as a guide aimed at desirable intake for helping achieve and maintain health and although the extent to which dietary guidelines represent the range of foods normally consumed could be argued, however, the inclusion of the legume food group is well supported in the scientific literature. Relevant health outcome studies have focused on glycaemic control, blood pressure, and chronic disease [1,2,3], and although a unified daily target is lacking [4], studies support the inclusion of legumes at a dose of at least 50 g/day [5]. Furthermore, the risk of all-cause mortality has been shown to decrease by ~16% with increasing intake of legumes up to 150 g/day [6], with one 50 g serving possibly resulting in a 10% risk reduction in all-cause mortality. Intake globally is less than half that target at 21 g/day [7]. The lowest intake was recorded in Uzbekistan, with less than 1 g/day for adults over 25 years, and the highest in Rwanda at 115.8 g/day according to the 2019 Global Burden of Disease Study [8]. Greater emphasis and perhaps repositioning of legumes in dietary guidelines may be required to encourage intake for health, environmental and economic benefits [9,10].

The economic benefits of pulses intake have been examined in Canada, but rather than 50 g/day, 100 g was modeled to suggest possible annual savings equal to Can$38–370 million in health care and related costs of type 2 diabetes- and cardiovascular disease combined [11]. Our previous nutrition economics research has assessed whole grains [12,13], but the combination with legumes is an important consideration, providing a complement of amino acids, which is important in plant-based and flexitarian dietary patterns. However, limiting the potential of legumes as a replacement for meat is also debated. In an examination of 94 food based dietary guidelines (FBDG), yet to be published, we found the classification of legumes highly variable, and while 38% of countries categorized legumes in the protein rich food group, 20% were in a group on their own and 15% were in the starchy staples group. Regardless of the categorization, the inclusion of legumes in FBDG is essential, and the specific phrasing even more so. The addition of legumes to the vegetable and the meat group is utilized in the Australian guidelines, however, the addition of ‘legumes and/or beans’ at the conclusion of each guideline statement is thought to do little in terms of inspiring intake [9].

As the body of evidence is relatively small for legumes, providing evidence of the health care cost savings based on the regular inclusion in diets could help guide decisions about the positioning and emphasis in dietary guidelines. Fifty grams per day, or 350 g over a week may be reasonably added to diets, considering that an international collaboration suggested a universal 100 g serve (or ½ cup) [4]. The aim of this study was to estimate the annual health care cost savings related to the inclusion of 50 g/day of legumes relevant to reductions in coronary heart disease (CHD) in adult Australians.

## 2. Materials and Methods

### 2.1. Study Design

A three-step cost-of-illness analysis was developed on the basis of (1) estimates of current per capita [8] and a targeted level of legumes intake [6] among Australian adults (age 25+ y), (2) estimates of percent reductions in relative risk (95% CI) of CHD following legumes intake [14], and (3) recent data on annual costs related to CHD management within the Australian health care system [15]. To assess the uncertainty factor, a sensitivity analysis of four scenarios (very pessimistic, pessimistic, optimistic, and universal) was conducted, as modelled previously [12,13]. Input parameters are summarized in Table 1.

### 2.2. Step 1: Employing Estimates of Current per Capita and Targeted Level of Legumes Intake

Any public health model that attempts to assess a potential benefit of healthy dietary patterns should consider the consumer’s perception and behavior in the marketplace and at the dining table. Based on the 2019 Global Burden of Disease data [8], in the first step of this analysis, the current estimated per capita legumes intake of 19.3 g/day for adults (age 25+ y) was compared to a targeted level of 50 g/day [6] and a calculation was built on estimates of proportions of Australian adults (age 25+ y) who are likely to increase their legumes intake by the gap amount of 30.7 g daily. As previously [12,13], here, the sensitivity analysis assumed very pessimistic, pessimistic, optimistic, and universal scenarios to represent 5%, 15%, 50%, and 100% (all) of prospective consumers who would reach the targeted daily level of legumes intake in the short term, short-to-medium-term, medium-to-long-term, and long-term, respectively.

### 2.3. Step 2: Establishing Percent Reductions in Relative Risk of Coronary Heart Disease with Legumes Intake

Only a few meta-analyses of prospective cohort studies have recently assessed the relationship between legumes intake and CHD as a hard endpoint. The majority of these studies only report data on highest vs. lowest intakes [16,17,18], with no specific serving sizes (i.e., g/day amounts) provided thereof, and some only examine specific types of legumes (e.g., soy) or legumes intake within the context of certain dietary patterns such as the Mediterranean diet [19]. It appears that at this stage the evidence on such a relationship, and related health benefits thereof, is moderate overall. Upon a keyword search for the relevant English-language literature on PubMed, in the second step of the analysis, the dose-response figures by Bechthold et al. [14] of ~10% reduction in CHD risk with 1 serving intake were utilized. The systematic review and dose-response meta-analysis of prospective studies included 123 reports (8 studies on non-linear dose-response association between legumes intake and CHD risk) and suggested a summary relative risk (RR) per 1 serving (~100 g/day) equal to 0.89 (95% CI 0.81–0.97) [14]. Similar rates of risk reduction were reported by other meta-analyses that only provided evidence on highest vs. lowest intakes [6,20,21]. Building on this while assuming a linear relationship, a 3.4% (95% CI 0.9–5.8) reduction in CHD risk per 30.7 g/day of legumes intake was established and utilized in the final step of the analysis.

### 2.4. Step 3: Calculalting Annual Savings in Direct Health Care Costs Related to Coronary Heart Disease in Australia

The third and final step of the analysis calculated the annual savings in CHD-related costs within the Australian health care system that would potentially follow the targeted level of legumes intake (Step 1) and the estimated reduction in CHD risk (Step 2). As previously described [12,13], the most recent estimates of direct health expenditure (the year 2018–2019) reported by the Australian Institute of Health and Welfare (AIHW) [15] were first inflated to the year 2022 equivalent levels, based on adjustment of rates according to the Australian Bureau of Statistics (ABS) Consumer Price Index (Health group) [22] (Table 2), and then utilized within arithmetic calculations where components of the cost categories were examined individually for assessment of savings, with 1% reduction in costs assumed to correspond to each 1% decrease in CHD risk. Additionally, as previously outlined [12,13], using the net present value equation, a 7% real discount rate was applied to the sum of savings in present day costs related to CHD management to assess the discounted value of different scenarios of legumes intake over a 20-year time frame at five-year increments after the year 2022 (year 0).

## 3. Results

Savings that could be predicted in CHD-related health care costs when legumes intake is increased from the current per capita level of 19.3 g/day to the 50 g/day target level across proportions of the Australian adult population are summarized in Table 3. Under the very pessimistic scenario, assuming a 5% uptake rate over the short term, our analysis predicted total health care savings equal to AUD 4.3 (95% CI 1.2–7.4) million in CHD cost annually. With a 15% uptake rate over the short-to-medium-term and a 50% uptake rate over the medium-to-long-term, the pessimistic and optimistic scenarios suggested cost savings equal to AUD 12.8 (95% CI 3.5–22.2) million and AUD 42.8 (95% CI 11.7–73.8) million, respectively. And, under the universal scenario, assuming a 100% uptake rate and long-term estimate of potential savings with the targeted increase in legumes intake, some total annual health care savings of AUD 85.5 (95% CI 23.3–147.7) million may be realized in avoided CHD costs.

As shown in Table 4, with a 7% discount rate, as per the Australian Governments recommendations [23], the sensitivity analysis of ‘very pessimistic’ through to ‘universal’ scenarios suggested total discounted savings in CHD-related health care costs of AUD 48.5 (95% CI 13.2–83.7) million to AUD 969.3 (95% CI 264.4–1674.3) million following the 50 g/day intake of legumes over a 20 year period (from 2022 through to 2041). Additionally, assuming adoption of each of the four scenarios every five years, i.e., the very pessimistic during years 0–4, pessimistic during years 5–9, optimistic during years 10–14, and universal scenario during years 15–19, the sum of total incremental discounted savings was estimated at AUD 290.2 (95% CI 79.1–501.3) million over the 20-year time frame.

## 4. Discussion

This cost-of-illness analysis supports a greater focus on legumes as a regular inclusion in the dietary pattern and demonstrates a predicted substantial savings for the Australian health care system in relation to costs for CHD from AUD 4.3 (95% CI 1.2–7.4) to AUD 85.5 (95% CI 23.3–147.7) million per year. Naturally, these figures are lower than the approximately $370 million in the Canadian study from 2017, which was based on an analysis of both type 2 diabetes and cardiovascular disease, at a level where 50% of the Canadian population would consume 100 g of legumes per day, in combination with a low glycaemic index or high fiber diet [11]. In consideration of the current Australian dietary pattern, and known lower current levels of consumption compared with Canada, the present economic analysis was based on the dose response data of 50 g (1/4 cup) of legumes per day by Schwingshackl et al. 2017 [6] who utilized the smallest serving with significant results for risk-decreasing foods in a systematic review and meta-analysis of prospective studies. The authors found that there was an absence of a linear association between legumes and all-cause mortality risk, with the nonlinear analysis showing ~16% reduced risk of all-cause mortality when consuming up to 150 g/day. However, this amount was considered unrealistic as a daily target for Australia, where the latest estimates from 2019 suggest 19.3 g/day [8]. Although 100 g/day was considered an unrealistically high daily target in light of current consumption and typical dietary patterns, we did calculate the potential yield savings in annual health care costs based on a target of 100 g of legumes per day, and thus a gap of 80.7 g/day compared to consumption estimates, as AUD 11.2 (95% CI 3.1–19.4) to AUD 224.8 (95% CI 61.3–388.3) million. This was more than double the savings predicted from reaching 50 g per day over time (Appendix A).

International serve size guidance has suggested 100 g or half a cup of cooked legumes [4], rather than 75 g (as a serving of vegetables) or 150 g (as a serving of lean meat) in the Australian Guide to Healthy Eating. However, this amount may still not be relevant as a daily target for Australians, although the amount (1/2 cup) could easily be consumed at a meal. The more reasonable target of 50 g/day (1/4 cup) indicates that our results may have further attributable savings, as consumers would not need to consume the food daily, yet could easily consume 350 g over one week. Increased consumption of legumes was supported through the dietary modeling performed for the purpose of informing the 2013 Australian Dietary Guidelines (ADGs), which recommends a 470% increase to meet the levels proposed [24].

Although determining the frequency of intake for legumes over a week presents more of a challenge than serving size, this analysis supports an achievable food volume for daily or weekly consumption. The main concern is setting a target being that amounts need to feasibly align with the cultural acceptability of a food, routine dietary patterns, and the infrastructure of the food systems [4,25]. Australian research points to consumers being receptive to increasing their intake, and “consumer attitudes towards legumes were positive, particularly in relation to their perceived health attributes” [10]. Others have found that knowledge of health benefits assisted consumers with planned dietary changes particularly “after reading the informational messages, 25–42% of all the participants said they planned to eat more legumes in the future” [26]. Favorable research has also been documented by Röös et al. (2022) [27] where “legumes were generally considered healthy and suitable in diets and many respondents stated an intention to increase consumption” and although they do not use them regularly, many consumers in New Zealand were also open to considering legumes as meat substitutes [28].

Legume consumption in the ADGs is promoted via the statements “consume plenty of vegetables, including different types and color, and legumes/beans” and “consume lean meat and poultry, fish, eggs, tofu, nuts and seeds, and legumes/beans” [29]. The inclusion of legumes as part of both the vegetable and meat alternatives groups aims to encourage visibility and consumption, however, there are equally valid concerns that legumes, in being included in both groups, do not give them the prominence they deserve. There have been suggestions that individualizing advice for the vegetable group, in particular for legumes, may improve consumer understanding and consumption [4,30,31]. A recent publication examined consumer preferences regarding the categorization and wording of both whole grain and legume statements in dietary guidelines (n = 314) [9]. When asked what would be helpful in achieving an increase in legume intake, the majority of participants preferred legumes to feature in their own food group (45%), and fewer suggested as part of the protein group (22%). When asked about wording, there was a significant preference for the statement “each day, consume at least one serve of legumes either as a serve of vegetables or as an alternative to meat” (*p* < 0.05). This statement provides both a specific frequency and quantification for legume consumption. Throughout the study, participants emphasized a preference for quantifiable recommendations expressed in cup measures, stating that “grams were less relevant and poorly visualized” [9].

In the planned revision of current ADGs, there has been a call to reflect environmental sustainability objectives as an opportunity to bring together environmental and population health goals [32,33]. A global shift towards plant-based foods, in their most natural form, would be considered beneficial for the health of humans and the planet as a dietary strategy [33,34]. Reflective of this, Canada’s dietary guidelines have recently shifted towards more of a plant-based diet, emphasizing and clearly depicting vegetables and fruits, whole grain food choices, and protein foods, including legumes together with meat, eggs, lower fat dairy, nuts, and seeds [35].

The changing food supply, increasing the number and type of plant-protein products, and legumes in other forms such as flour, pasta, snack food, bread products, convenient lunch portions, alternative packaging to traditional canned, and dried forms [36] may do more to stimulate intake than government guidelines per se. When used as an ingredient though, legumes within the dietary pattern become far more difficult to identify and distinguish in intake studies, even though complex methods of data collection are used at the national level. Plant-based meats are another obvious opportunity for legumes, however, the value of extracted protein from legumes, would need to be evaluated in comparison to the research supporting whole legume intake, as the nutrient profiles in these more processed foods are significantly altered. Research to date has found that of 137 plant-based meat alternatives available on Australian supermarket shelves only 4% were low in sodium (58–1200 mg/100 g), and less than a quarter of products (24%) were fortified with vitamin B_12_, 20% with iron, and 18% with zinc [37].

The analysis presented is based on a data-driven approach utilized previously [11,12,13], where nationally representative data was utilized, increasing the validity of the results. However, we were aware that the cost data was from 2018–2019, so this was inflated to accommodate the age of the data. We also utilized a discounted method [38] to accommodate time, as reported by the Australian Government. A meta-analysis of prospective data pertaining to CHD was used in preference to a similar study of randomized controlled trials in order to obtain overall disease outcomes. The risk reduction ranged from our chosen reference utilized (RR: 0.81–0.97), which approximates the RR from a meta-analysis of RCTs based on the Mediterranean diet and Cardiovascular Disease (CVD), where legumes were one of four dietary components with the most positive effects along with olive oil, vegetables and fruit (RR: 0.91; 95% CI: 0.83, 0.98; *I*^2^ = 33%) [19]. It is important to note that our analysis assumed a linear relationship that has not been shown to occur in nature [6]. Although changes in diet would not be immediate in terms of disease reduction, periods of one to three years lag time could be expected before individual or population benefits would be realized [39]. Adjustment for this time lag was not considered in our model, however, policy makers should not be dissuaded and view dietary change positively in light of the potential cost savings that could be realized through such very small changes. Finally, our sensitivity analysis was based on population-based adoption over time and utilized the reported risk reduction range from Bechtold et al. [14], however, effect sizes from observational studies have been shown to be more generous than randomized controlled trials [40], although these tend to report biomarkers rather than disease outcomes. This should be kept in mind when interpreting the analysis presented.

## 5. Conclusions

Nutrition economic analyses provide key support to directing efforts of population-based initiatives in relation to nutrition, dietary patterns, and health outcomes. The analyses combine metrics relevant to the specific population, incorporating known measures of consumption, and known health care costs, together with published risk reduction evidence, providing logical support and guidance for policy. This analysis is limited to capturing the impact of just one food type on one disease with substantial predicted savings annually and supports earlier work by our group on other key food groups. The potential economic benefits of incorporating legumes within the dietary patterns of Australians should provide greater impetus to promote this food group, consumed as part of a diet low in saturated fat and high in dietary fiber, in a more overt manner as part of revised dietary guidelines. But for Australians, this food could also assist with achieving broader sustainability measures in relation to diet, helping to bring together the environment and health as an important pillar in relation to sustainability. Although the plant-based protein movement seeks to view legumes in terms of nutrient components, protein, and perhaps dietary fiber, it is our assertion that food synergy, and the complex relationships within the food matrix, mean that the legume would be ideally consumed whole. Whole legume products are already plentiful within the Australian food supply in dried, frozen, canned, oven roasted, and as the main ingredient in dips.

## Figures and Tables

**Table 1:** Summary of the cost-of-illness analysis input parameters and corresponding references.

Parameter	Men and Women	Reference
Current per capita legumes intake, g/day	19.3	Global Burden of Disease Study [8]
Target legumes intake, g/day	50	Schwingshackl et al. [6]
Gap amount, g/day	30.7	
Proportions of prospective consumers ^1^	5%, 15%, 50%, 100%	Estimates
CHD relative risk (95% CI) per 100 g/day legumes intake, no. of studies	0.89 (0.81–0.97), n = 10	Bechthold et al. [14]
CHD% risk reduction (95% CI) per 30.7 g/day legumes intake ^2^	−3.4% (0.9–5.8)	

Abbreviations: CHD, coronary heart disease. ^1^ Estimates of proportions of Australian adults (age 25+ y) who would increase their current estimated per capita legumes intake (19.3 g/day) to the targeted level of 50 g/day over the short term (very pessimistic), short-to-medium term (pessimistic), medium-to-long term (optimistic), and long term (universal) scenarios. ^2^ Percent risk reduction (95% CI) per 30.7 g/day was calculated based on the summary relative risk (95% CI) values per 100 g/day by Bechthold et al. [14] assuming a linear relationship.

**Table 2:** Summary of coronary heart disease direct health expenditures in Australia (AUD million), age 25+ y population ^1^.

	Coronary Heart Disease	
	2018–19	2022 ^2^
Direct health expenditure		
Allied health and other services	1.8	1.9
General practitioner services	71.6	77.6
Medical imaging	23.3	25.3
Pathology	21.2	23.0
Pharmaceutical benefits scheme	155.1	168.2
Private hospital services	892.2	967.2
Public hospital admitted patient	823.0	892.2
Public hospital emergency department	103.8	112.5
Public hospital outpatient	142.6	154.6
Specialist services	101.2	109.7
**All areas**	**2335.8**	**2532.1**

Abbreviations: AUD, Australian dollar. ^1^ From the Australian Institute of Health and Welfare (AIHW) disease expenditure database (2018–2019) [15]. ^2^ Current dollars based on adjustment of inflation rates according to the Australian Bureau of Statistics (ABS) Consumer Price Index (Health group) [22].

**Table 3:** Potential annual savings in direct health expenditures of coronary heart disease in Australian adults (age 25+ y) with 50 g/day legumes intake (AUD million) ^1^.

	Scenario			
	Very Pessimistic	Pessimistic	Optimistic	Universal
Direct health expenditure savings				
Allied health and other services	<0.1 (<0.1–<0.1)	<0.1 (<0.1–<0.1)	<0.1 (<0.1–0.1)	0.1 (<0.1–0.1)
General practitioner services	0.1 (<0.1–0.2)	0.4 (0.1–0.7)	1.3 (0.4–2.3)	2.6 (0.7–4.5)
Medical imaging	<0.1 (<0.1–0.1)	0.1 (<0.1–0.2)	0.4 (0.1–0.7)	0.9 (0.2–1.5)
Pathology	<0.1 (<0.1–0.1)	0.1 (<0.1–0.2)	0.4 (0.1–0.7)	0.8 (0.2–1.3)
Pharmaceutical benefits scheme	0.3 (0.1–0.5)	0.9 (0.2–1.5)	2.8 (0.8–4.9)	5.7 (1.5–9.8)
Private hospital services	1.6 (0.4–2.8)	4.9 (1.3–8.5)	16.3 (4.5–28.2)	32.7 (8.9–56.4)
Public hospital admitted patient	1.5 (0.4–2.6)	4.5 (1.2–7.8)	15.1 (4.1–26.0)	30.1 (8.2–52.0)
Public hospital emergency department	0.2 (0.1–0.3)	0.6 (0.2–1.0)	1.9 (0.5–3.3)	3.8 (1.0–6.6)
Public hospital outpatient	0.3 (0.1–0.5)	0.8 (0.2–1.4)	2.6 (0.7–4.5)	5.2 (1.4–9.0)
Specialist services	0.2 (0.1–0.3)	0.6 (0.2–1.0)	1.9 (0.5–3.2)	3.7 (1.0–6.4)
**All areas**	**4.3 (1.2–7.4)**	**12.8 (3.5–22.2)**	**42.8 (11.7–73.8)**	**85.5 (23.3–147.7)**

Abbreviations: AUD, Australian dollar. ^1^ Data (95% CI) are potential monetary savings following coronary heart disease risk reduction with 50 g/day intake of legumes (Table 1). The very pessimistic, pessimistic, optimistic, and universal scenarios are modeled to represent short term, short-to-medium-term, medium-to-long-term, and long-term of estimates of potential savings in CHD-related health care costs that could follow when, respectively, 5%, 15%, 50%, and 100% of Australian adults (age 25+ y) consume the targeted daily level of legumes.

**Table 4:** Sum of potential total discounted savings on direct health care expenditures of coronary heart disease in Australian adults (age 25+ y) with 50 g/day legumes intake over short- and long-term periods (AUD million) ^1^.

	Scenario			
	Very Pessimistic	Pessimistic	Optimistic	Universal
Years 0 to 4	18.8 (5.1–32.4)	56.3 (15.3–97.2)	187.6 (51.2–324.0)	375.2 (102.3–648.0)
Years 5 to 9	13.4 (3.6–23.1)	40.1 (10.9–69.3)	133.7 (36.5–231.0)	267.5 (72.9–462.0)
Years 10 to 14	9.5 (2.6–16.5)	28.6 (7.8–49.4)	95.4 (26.0–164.7)	190.7 (52.0–329.4)
Years 15 to 19	6.8 (1.9–11.7)	20.4 (5.6–35.2)	68.0 (18.5–117.4)	136.0 (37.1–234.9)
**Total discounted savings**	**48.5 (13.2–83.7)**	**145.4** **(39.7–251.1)**	**484.7** **(132.2–837.1)**	**969.3** **(264.4–1674.3)**

Abbreviations: AUD, Australian dollar. ^1^ Data (95% CI) are potential total discounted monetary savings following coronary heart disease risk reduction with 50 g/day intake of legumes. The very pessimistic, pessimistic, optimistic, and universal scenarios are modeled to represent short term, short-to-medium-term, medium-to-long-term, and long-term of estimates of potential savings in CHD-related health care costs that could follow when, respectively, 5%, 15%, 50%, and 100% of Australian adults (age 25+ y) consume the targeted daily level of legumes.

## Data Availability

All data for this study is contained within the article.

## Appendix A

**Table A1:** Potential annual savings in direct health expenditures of coronary heart disease in Australian adults (age 25+ y) with 100 g/day legumes intake (AUD million) ^1^.

	Scenario			
	Very Pessimistic	Pessimistic	Optimistic	Universal
Direct health expenditure savings				
Allied health and other services	<0.1 (<0.1–<0.1)	<0.1 (<0.1–<0.1)	0.1 (<0.1–0.1)	0.2 (<0.1–0.3)
General practitioner services	0.3 (0.1–0.6)	1.0 (0.3–1.8)	3.4 (0.9–5.9)	6.9 (1.9–11.9)
Medical imaging	0.1 (<0.1–0.2)	0.3 (0.1–0.6)	1.1 (0.3–1.9)	2.2 (0.6–3.9)
Pathology	0.1 (<0.1–0.2)	0.3 (0.1–0.5)	1.0 (0.3–1.8)	2.0 (0.6–3.5)
Pharmaceutical benefits scheme	0.7 (0.2–1.3)	2.2 (0.6–3.9)	7.5 (2.0–12.9)	14.9 (4.1–25.8)
Private hospital services	4.3 (1.2–7.4)	12.9 (3.5–22.2)	42.9 (11.7–74.1)	85.9 (23.4–148.3)
Public hospital admitted patient	4.0 (1.1–6.8)	11.9 (3.2–20.5)	39.6 (10.8–68.4)	79.2 (21.6–136.8)
Public hospital emergency department	0.5 (0.1–0.9)	1.5 (0.4–2.6)	5.0 (1.4–8.6)	10.0 (2.7–17.3)
Public hospital outpatient	0.7 (0.2–1.2)	2.1 (0.6–3.6)	6.9 (1.9–11.8)	13.7 (3.7–23.7)
Specialist services	0.5 (0.1–0.8)	1.5 (0.4–2.5)	4.9 (1.3–8.4)	9.7 (2.7–16.8)
**All areas**	**11.2 (3.1–19.4)**	**33.7 (9.2–58.2)**	**112.4 (30.7–194.1)**	**224.8 (61.3–388.3)**

Abbreviations: AUD, Australian dollar. ^1^ Data (95% CI) are potential monetary savings following coronary heart disease risk reduction with 100 g/day intake of legumes. The very pessimistic, pessimistic, optimistic, and universal scenarios are modeled to represent short term, short-to-medium-term, medium-to-long-term, and long-term of estimates of potential savings in CHD-related health care costs that could follow when, respectively, 5%, 15%, 50%, and 100% of Australian adults (age 25+ y) consume the targeted daily level of legumes.

**Table A2:** Sum of potential total discounted savings in direct health care expenditures of coronary heart disease in Australian adults (age 25+ y) with 100 g/day legumes intake over short- and long-term periods (AUD million) ^1^.

	Scenario			
	Very Pessimistic	Pessimistic	Optimistic	Universal
Years 0 to 4	**49.3 (13.4–85.2)**	147.9 (40.3–255.5)	493.1 (134.5–851.7)	986.2 (269.0–1703.4)
Years 5 to 9	35.2 (9.6–60.7)	**105.5 (28.8–182.2)**	351.6 (95.9–607.2)	703.1 (191.8–1214.5)
Years 10 to 14	25.1 (6.8–43.3)	75.2 (20.5–129.9)	**250.7 (68.4–432.9)**	501.3 (136.7–865.9)
Years 15 to 19	17.9 (4.9–30.9)	53.6 (14.6–92.6)	178.7 (48.7–308.7)	**357.4 (97.5–617.4)**
**Total discounted savings**	**127.4** **(34.7–220.1)**	**382.2** **(104.2–660.2)**	**1274.0** **(347.5–2200.5)**	**2548.0** **(694.9–4401.1)**
**Total incremental discounted savings with adoption of each scenario every 5 years (years 2022–2041)**				**762.9** **(208.1–1317.7)**

Abbreviations: AUD, Australian dollar. ^1^ Data (95% CI) are potential total discounted monetary savings following coronary heart disease risk reduction with 100 g/day intake of legumes. The very pessimistic, pessimistic, optimistic, and universal scenarios are modeled to represent short term, short-to-medium-term, medium-to-long-term, and long-term of estimates of potential savings in CHD-related health care costs that could follow when, respectively, 5%, 15%, 50%, and 100% of Australian adults (age 25+ y) consume the targeted daily level of legumes.
